# ID 47 - Caracterização Técnica de Profissionais Atuando em ATS no Âmbito Estadual de Saúde: dados preliminares

**DOI:** 10.5327/2237-9622.2025.v34s1.47

**Published:** 2025-11-25

**Authors:** Barbara Cristiane da Silva, Bruna Marmett, Ana Paula Blankenheim, Rodrigo Pereira de Almeida, Roseana Boek Carvalho, Marina Petrasi Guahnon, Muriel Primon de Barros, Airton Tetelbom Stein, Cinara Stein, Gilson Pires Dorneles, Suena Medeiros Parahiba, Maicon Falavigna

**Keywords:** saúde baseada em evidência, secretarias estaduais de saúde, Avaliação de Tecnologia em Saúde, tomada de decisão

## Abstract

**Introdução:**

O número de profissionais que atuam em Avaliação de Tecnologias em Saúde (ATS) no Brasil tem aumentado nos últimos anos devido ao avanço da área. A implementação efetiva dos processos de ATS requer a atuação de profissionais com habilidades e conhecimentos específicos. No âmbito das Secretarias Estaduais de Saúde (SES), a ATS é um tema incipiente e não há mapeamento do nível de conhecimentos em ATS dos profissionais. Sendo de extrema importância capacitar profissionais das SES na área de ATS, particularmente em um ecossistema de limitação de recursos na área do setor saúde. Portanto, objetivamos realizar a caracterização profissional de servidores estaduais que atuam na área de ATS nas SES.

**Método:**

O estudo apresenta um delineamento observacional com o objetivo de mapear as características técnicas e de percepções sobre prática de saúde baseada em evidências (SBE). Foi enviado um formulário para os profissionais que atuam em ATS em todas as SES do Brasil contemplando questões de caracterização técnica e perguntas do Instrumento para Avaliação da Saúde Baseada em Evidências (I-SABE). As primeiras seis seções incluem questões referentes aos seguintes dados: formação acadêmica; produção científica; conhecimentos gerais; dedicação à ATS. Posteriormente, seguem as questões referentes à percepção sobre saúde baseada em evidência. O formulário foi enviado no formato de Google Forms. Os dados preliminares foram analisados de forma descritiva e apresentados como frequência absoluta e relativa. O projeto de pesquisa foi aprovado pelo Comitê de Ética em Pesquisa (CEP) do Hospital Moinhos de Vento sob número de Parecer 6.963.930.

**Resultados:**

Até o momento da análise de dados, 17 profissionais de nove diferentes SES haviam respondido ao questionário. A carga horária semanal média de trabalho na SES era de 34 horas, sendo que 45% da carga horária era dedicada às atividades de ATS. Oito profissionais estavam vinculados a um Nats e tinham uma média de dedicação mensal de 12 horas. Entre os respondentes, 4 (24%) tinham mais de cinco anos de experiência com ATS, 1 (6%) tinha pós-doutorado, 3 (18%) doutorado, 10 (59%) tinham mestrado acadêmico ou profissional e 3 (18%) tinham especialização. De acordo com o relato dos profissionais, o conhecimento em temas como parecer técnico científico, revisão sistemática e metanálise é intermediário. O nível de conhecimento foi relatado como básico ou ausente para os tópicos metanálise em rede, avaliação da certeza da evidência pelo Sistema GRADE, modelagem em avaliação de simulação de eventos discretos. Mais de 90% dos profissionais sentem-se capazes em utilizar a SBE, 88% usam SBE para orientar suas decisões, 81% consideram que a SBE os fazem sentir confiança na decisão clínica e engloba sua experiência profissional, e 92% acreditam que a prática da SBE afeta positivamente a decisão clínica.

**Conclusão:**

O estudo mostrou que existe uma grande variação de conhecimento entre os profissionais das SES que atuam em ATS. No entanto, não há dedicação exclusiva a estas atividades. De forma geral, os profissionais compreendem a importância e aplicam SBE nas suas decisões.

